# Comparison of six commercial kits to extract bacterial chromosome and plasmid DNA for MiSeq sequencing

**DOI:** 10.1038/srep28063

**Published:** 2016-06-17

**Authors:** Laura Becker, Matthias Steglich, Stephan Fuchs, Guido Werner, Ulrich Nübel

**Affiliations:** 1 Robert Koch Institute, FG13 Division of Nosocomial Pathogens and Antibiotic Resistances, Department of Infectious Diseases, Wernigerode, Germany; 2 Leibniz Institute DSMZ, Braunschweig, Germany; 3 German Centre of Infection Research (DZIF), Partner Site Hannover-Braunschweig, Braunschweig, Germany

## Abstract

We compared commercial kits for extraction of genomic DNA from the Gram-negative bacterium *Klebsiella pneumoniae* for subsequent Miseq sequencing. Purification of DNA was based on matrix binding (silica or anion exchange resin) or differential precipitation (salting out), respectively. The choice of extraction kit had little effect on sequencing quality and coverage across drastically different replicons, except for an apparent depletion of small plasmids (<5 kb) during precipitation-based extractions. Sequencing coverage provided copy-number estimates for small plasmids that were consistently higher than those from quantitative real-time PCR.

Recently, DNA sequencing technologies have undergone major improvements in terms of sequencing speed, throughput, and associated costs^1^. Due to this development, ‘next generation’ sequencing is about to be integrated into routine practice in clinical microbiology laboratories^2^,^3^. Bacterial whole-genome sequencing in the diagnostic context enables pathogen identification and strain genotyping with ultimate discriminatory power for detection of transmission chains and outbreaks^4^,^5^. Furthermore, it promises to enable the prediction of a microbe’s phenotype, including antibiotic resistance and virulence^6^,^7^,^8^.

For preparation of microbial DNA for sequencing, robust extraction methods are required. Commercially available DNA extraction kits are usually preferred, as they provide superior reproducibility, quality control, and potential for automation. These kits rely on different principles for DNA purification, including solution- and solid-phase-based protocols^9^. While the latter make use of DNA-adsorbing materials (e.g. silica-membranes, silica-covered magnetic beads, or anion-exchange columns), which specifically bind DNA and subsequently release it to an appropriate buffer, solution-based (salting out) protocols are based on precipitation of DNA. The purity of DNA was previously reported to have effects on the reproducibility of sequencing library preparations^10^ and on the evenness of sequencing read distribution along the sequenced genome^11^. Moreover, depending on the extraction method applied, the purification of DNA molecules is known to be influenced by their specific size, nucleotide composition, topology, and association with proteins^12^,^13^. Since individual bacterial genomes frequently consist of several replicons that may vary in size and copy number by orders of magnitude (e. g., chromosomes, plasmids), differential extraction efficiency and unequal sequence representation may be expected. For compensation, costly increased overall sequencing coverage may be required to ensure reliable detection of diagnostically relevant polymorphisms and genes (e. g., predictive markers for antimicrobial resistance).

Numerous studies have documented that PCR-based analyses of microbial community composition may be affected by the DNA extraction methods applied, due to their differential efficiency for diverse microorganisms (for recent examples, see^14^,^15^,^16^,^17^). In contrast, comparative analyses of DNA extraction protocols for (meta-)genomic investigations or diagnostics are scarce^18^. To our best knowledge, the suitability of DNA extraction kits relying on different technical principles for purification of DNA from bacterial cultures to be used in genomic sequencing has not been systematically assessed.

In the present study, we compared the performance of six commercially available kits for extraction of DNA, namely Genomic-tip 20/G, MagAttract HMW DNA Kit, MasterPure DNA Purification Kit, Wizard Genomic DNA Purification Kit, DNeasy Blood & Tissue Kit and Plasmid Mini Kit, for subsequent Illumina Miseq sequencing. Experiments were performed using a clinical *Klebsiella pneumoniae* isolate, whose fully sequenced genome consists of a 5,278 kb chromosome, one large plasmid (362 kb), and two small plasmids (4.8 kb and 3.8 kb)^4^,^19^.

## Characteristics of the kits compared

*Klebsiella pneumoniae* 234–12 was inoculated into 40 ml of brain-heart-infusion broth and incubated at 37 °C and shaking at 140 rpm for 15 hours, resulting in 4 × 10^9^ bacterial cells per milliliter. This culture was aliquoted and the biomass was pelleted by centrifugation and stored at −20 °C prior to DNA extraction. Basic characteristics of the kits compared are listed in Table 1. Extraction costs per sample varied from € 1.10 to € 8.10, with salting-out kits being the least expensive (Table 1). Extractions were performed according to the manufacturers’ protocols, and DNA was eluted or redissolved, respectively, in nuclease free water. All kits required similar hands-on time (35–60 minutes for three samples), but the lengths of incubation periods and total completion times varied more widely (Table 1).

## Effect of DNA extraction kit on DNA quality

**Figure 1:**
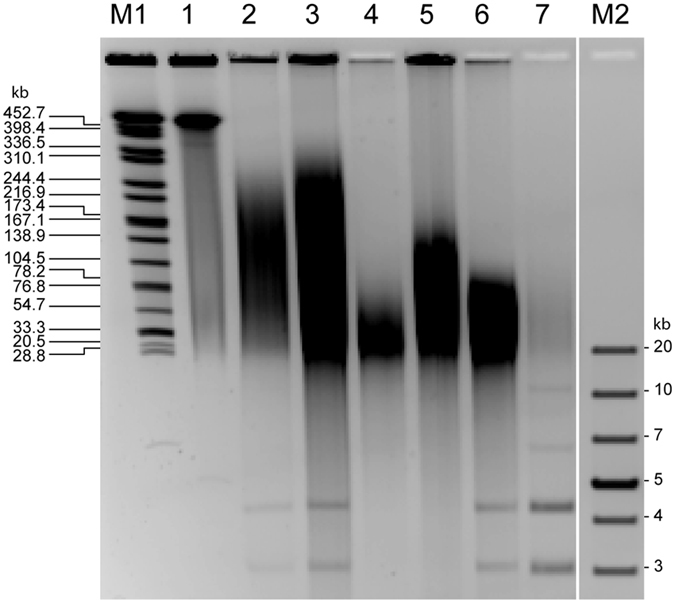
Pulsed-field gel electrophoresis of *K. pneumoniae* 234-12 DNA extracted with six different kits. DNA was extracted from aliquots of the same overnight culture according to the manufacturers′ protocols, and 10 μl of resulting extracts were loaded per lane. M1 Size standard *Salmonella* Braenderup lysed in agarose plug, DNA digested with XbaI^27^. 1 *K. pneumoniae* 234-12 lysed in agarose plug^28^, DNA digested with S1 nuclease for presentation of the linearized 362-kb plasmid^29^. 2 Genomic-tip 20/G. 3 MagAttract HMW DNA Kit. 4 MasterPure DNA Purification Kit. 5 Wizard Genomic DNA Purification Kit. 6 DNeasy Blood & Tissue Kit. 7 Plasmid Mini Kit. M2 GeneRuler 1 kb Plus DNA Ladder (Thermo Scientific).

While DNA extracted with the Genomic-tip, MasterPure and MagAttract kits met the A260/A280 absorbance ratio (1.8–2.0) recommended for preparation of Nextera XT libraries (Illumina)^20^, other kits deviated from this range (Table 1). DNA yields determined by using an assay based on fluorescence (PicoGreen, Molecular Probes) varied considerably (Table 1), but all kits supplied sufficient amounts of DNA (i. e., ≥1 ng) for Nextera XT library preparation. Pulsed-field gel electrophoresis (PFGE) indicated that the MagAttract and Genomic-tip kits provided the largest DNA fragments (up to 300 kb) (Fig. 1). Accordingly, the 362-kb plasmid was not visible as a distinct band in any of the DNA extracts (Fig. 1). In contrast, the two small plasmids were visually detectable as bands of 4 and 5 kb, respectively, in DNA extracts from all kits applying binding of DNA to some matrix (Fig. 1).

## Quality characteristics of sequencing libraries

Sequencing libraries were prepared using the Nextera XT kit (Illumina) according to the manufacturer’s protocol. Capillary electrophoresis (applying the High Sensitivity DNA kit on an Agilent 2100 Bioanalyzer) did not reveal any fragment-size differences between libraries prepared from the different extracts (not shown). Libraries were sequenced on a MiSeq machine (Illumina) using v3 reagents with 2 × 300 cycles according to the manufacturer’s instructions. In resulting sequencing reads, 75 to 89% of bases had quality scores ≥Q30, and this proportion was independent from the DNA extraction method (data not shown). Sequencing reads were aligned to the reference genome sequence (concatenated chromosomal and plasmid sequences, accession nos. CP011313 to CP011316) by using BWA-SW (version 0.7.12-r1039, default parameters^21^). Read alignments (BAM files processed with SAMtools^22^) did not reveal any significant differences in read length and read span (insert size) between extraction kits (determined using a Python script^23^, no significant difference compared to the DNeasy kit, p ≥ 0.02; data not shown).

## Sequencing coverage of chromosome and plasmid DNA

**Figure 2:**
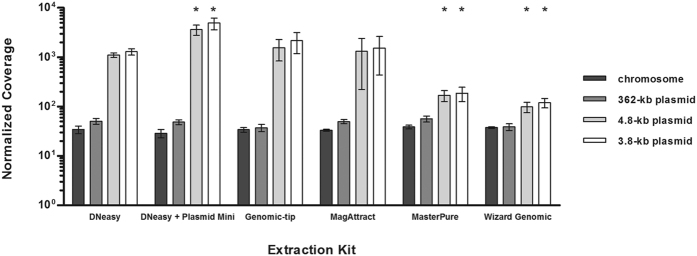
Effect of DNA extraction kit on the sequencing coverage of the chromosome and the three plasmids of *K. pneumoniae* 234–12. Means and standard deviations from three independent experiments are reported. For statistical analysis, two-tailed student’s t-tests with Bonferroni correction were performed (global significance level α = 0.10). Asterisks indicate a statistically significant difference compared to the DNeasy kit (p-value below 0.02). One gene was excluded from this evaluation, because orthologues were found on both the chromosome and the 3.8-kb plasmid.

**Figure 3:**
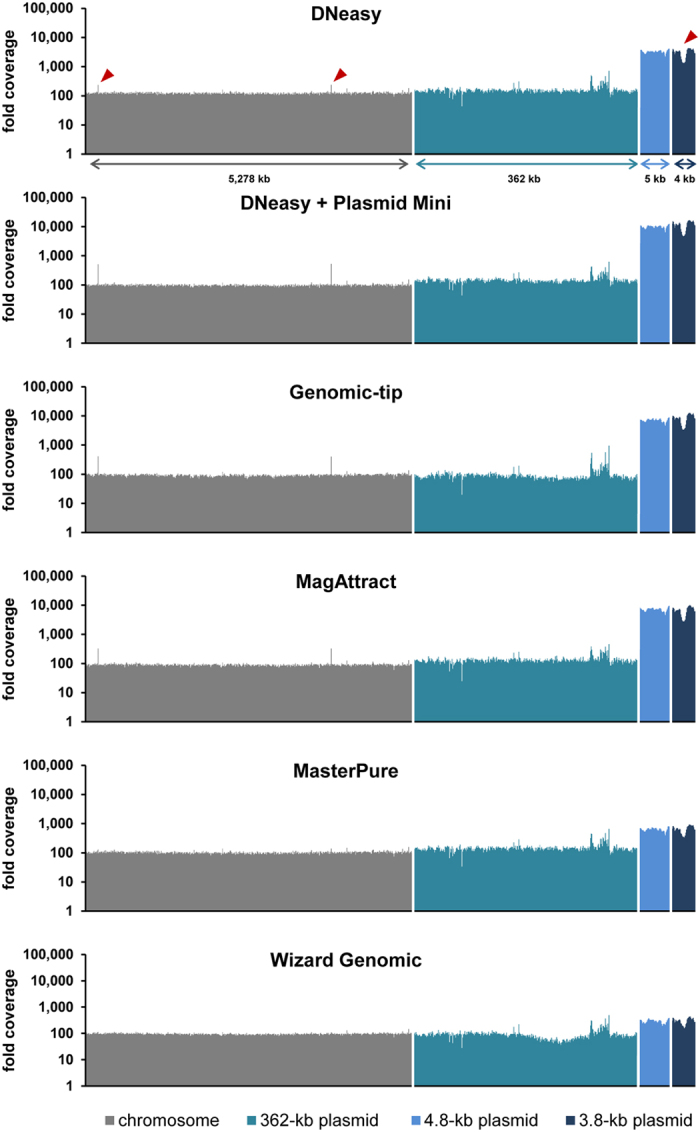
Coverage along replicons. Bars correspond to the mean coverage of 10,000 nucleotides for the chromosome, 1,000 nucleotides for the 362-kb plasmid and 100 nucleotides for the two small plasmids, respectively. Pointed regions (red arrows) contain a gene occurring on both the 3.8-kb plasmid and chromosome.

Sequencing coverage along the reference genome sequence was determined from the read alignment by applying the sequence viewer module in Geneious 7.1.4 software^24^ and normalized for the overall number of reads per library (Fig. 2). All DNA extraction kits resulted in 29 to 56-fold average coverage of the chromosome and the 362-kb plasmid (Fig. 2; no significant difference compared to the DNeasy kit, p ≥ 0.02). Further, the two small plasmids achieved higher coverage than the chromosome in all cases (Fig. 2); for example, based on the Genomic-tip extract, the mean coverage was 34× (standard deviation, 3.7) for the chromosome and 2,164× (standard deviation, 982) for the 3.8-kb plasmid (Fig. 2). Strikingly, however, DNA extraction with salting-out kits (MasterPure, Wizard Genomic) when compared to other kits resulted in 7–12 fold lower coverage of both small plasmids (p < 0.02; Fig. 2). The true ratio of plasmid and chromosome copy numbers within the bacterial cells cannot easily be determined. By using quantitative real-time PCR^25^ (qPCR), we estimated that the two small plasmids were 2–3 times more abundant than the chromosome in a crude extract (based on boiling a resuspended bacterial cell pellet for 10 min. and subsequent centrifugation to remove cell debris), and 3–9 times more abundant than the chromosome in matrix-based extracts (Suppl. Fig. S1). The slight enrichment of small plasmids may be caused by their preferential binding to anion exchange resins or silica matrices^12^,^13^. In contrast, small plasmids apparently got depleted during extractions with salting-out kits, since their copy numbers were estimated by qPCR to be lower than in the crude extract and even lower than that of the chromosome (down to only 20%; Suppl. Fig. S1). Interestingly, copy numbers of small plasmids appeared almost ten-fold higher based on sequencing coverage results when compared to qPCR results (Fig. 3, Suppl. Fig. S1). Since it was previously reported that qPCR may underestimate the copy number of supercoiled plasmids in contrast to molecules linearized by restriction digestion^26^, we assume that our sequencing coverage results provide more precise estimates of actual copy numbers. Independent from the DNA extraction kits used, sequencing coverage was very even along each of the replicons (Fig. 3). In any case, all kits yielded sufficient coverage for all replicons. Hence, the more balanced coverage of chromosome and plasmids achieved with salting-out protocols may be considered advantageous for economic reasons, as smaller overall sequencing output is required.

## Conclusion

In conclusion, all DNA extraction kits tested yielded satisfactory MiSeq sequencing results. Our investigation was limited to a single bacterial isolate and species, which prohibits wide generalization. However, it was notable that the choice of extraction kit had little effect on sequencing read quality and on the evenness of sequencing coverage. In cases where a differential coverage of smaller plasmids (<5 kb in our case) may be considered negligible, the choice of DNA extraction kit can be guided largely by other factors including extraction costs, extraction time and potential for automation.

## Additional Information

**Accession codes:** Sequence data were submitted to the European Nucleotide Archive (http://www.ebi.ac.uk/ena) under accession number PRJEB10820.

**How to cite this article**: Becker, L. *et al.* Comparison of six commercial kits to extract bacterial chromosome and plasmid DNA for MiSeq sequencing. *Sci. Rep.* **6**, 28063; doi: 10.1038/srep28063 (2016).

## Supplementary Material

Supplementary Information

## Figures and Tables

**Table 1:** Summary of DNA extraction kit characteristics.

Extraction Method	Manufacturer	Principle	Costs per sample*[€]	Completion time*(hands-on-time)	Cell count	Yield [μg] (SD)	Purity [A260/280] (SD)
Genomic-tip 20/G	Qiagen	anion-exchange column (gravity)	8.1	8 h (45 min)	4 × 10^9^	9.8 (3.5)	1.77 (0.06)
MagAttract HMW DNA Kit	Qiagen	DNA-binding magnetic beads, silica-based	4.4	2 h 40 min (1 h)	2 × 10^9^	10.3 (6.6)	1.83 (0.05)
MasterPure DNA Purification Kit	Epicentre	salting-out	1.1	2 h 10 min (35 min)	0.4 × 10^9^	3.3 (1.0)	1.82 (0.03)
Wizard Genomic DNA Purification Kit	Promega	salting-out	2.0	3 h (35 min)	4 × 10^9^	18.1 (7.5)	1.58 (0.01)
DNeasy Blood & Tissue Kit	Qiagen	silica-membrane column (spin)	3.2	3 h (45 min)	2 × 10^9^	10.9 (1.3)	1.72 (0.05)
Plasmid Mini Kit	Qiagen	alkaline lysis, anion-exchange column (gravity)	5.2	3 h 50 min (40 min)	18 × 10^9^	0.50 (0.2)	1.67 (0.03)

^*^List price on manufacturer web page in July 2015.

^**^Approximate time to complete DNA extraction from three samples.

SD: standard deviation from three independent DNA extractions.
