# Supplementary material for: Comparison of six commercial kits to extract bacterial chromosome and plasmid DNA for MiSeq sequencing
Source: Sci Rep. 2016 Jun 17;6:28063. doi: 10.1038/srep28063 (PMC4911584; doi:10.1038/srep28063)
Supplement: Supplementary Information [file srep28063-s1.doc]

**Comparison of six commercial kits to extract bacterial chromosome and plasmid DNA for MiSeq sequencing**

**-- Supplementary material** --

Laura Becker,^a^ Matthias Steglich,^b^ Stephan Fuchs, ^a^ Guido Werner,^a^ Ulrich Nübel^b,c^*

^a^ Robert Koch Institute, FG13 Division of Nosocomial Pathogens and Antibiotic Resistances, Department of Infectious Diseases, Wernigerode Branch, Germany;

^b^ Leibniz Institute DSMZ, Braunschweig, Germany;

^c^ German Centre of Infection Research (DZIF), Partner Site Hannover-Braunschweig, Braunschweig, Germany

**
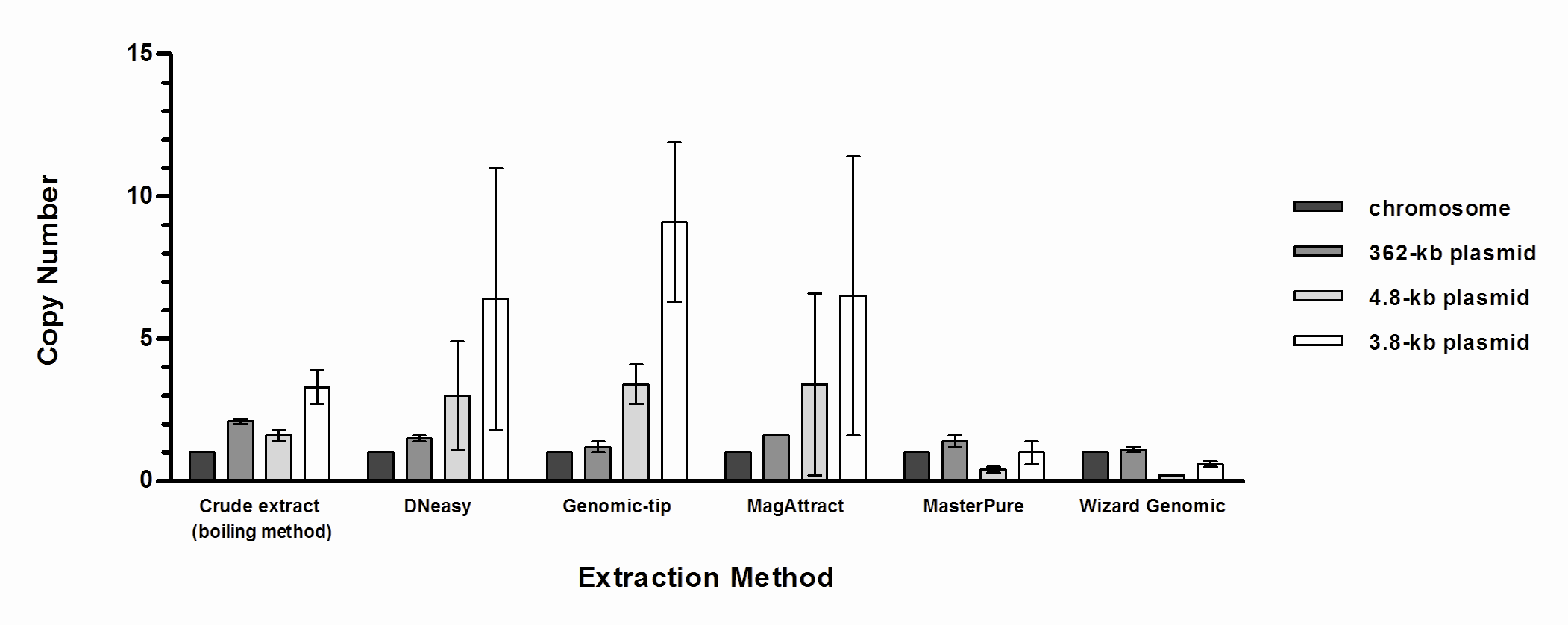
**

**Supplementary Figure S1**. Plasmid copy numbers in DNA extracts determined by qPCR. Means and standard deviations from three independent experiments are reported. All qPCR experiments were performed on a Biorad CFX96 C1000 machine using the DyNAmo ColorFlash SYBR Green qPCR Kit (Thermo Scientific) according to the manufacturer’s instructions and specific primer pairs for each replicon (Supplementary Table S1). The PCR protocol included an incubation at 95.0° C for 7 min, followed by 40 cycles of 95.0° C for 10 s, 55.0° C for 15 s, 72.0° C for 30 s and subsequent plate reading. Primer efficiencies (*E*) were calculated from the slope of standard curves based on dilution series for DNA extracted with the boiling method and the Genomic-tip kit, respectively (formula 1). The plasmid copy number was defined as the number of plasmid copies per chromosome. Plasmid copy numbers (*PCN*) were calculated from *Ct* (threshold cycle) values using formula 2 (*C* – chromosome, *P* – plasmid)^1^.

1.
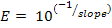

2.
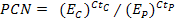


**Reference.**

1 Skulj, *M. et a*l. Improved determination of plasmid copy number using quantitative real-time PCR for monitoring fermentation processes*. Microb Cell Fa*c**t** 7, 6, doi:10.1186/1475-2859-7-6 (2008).

| **Replicon** | **Primer name** | **Sequence (5‘ – 3‘)** | **Position** | **Target** | **Product size** |
| --- | --- | --- | --- | --- | --- |
| Chromosome | Chromosome-F | TCACTTCCGGGTCGTCAATG | 4,189,670 – 4,189,689 | crl gene | 100 bp |
|  | Chromosome-R | ATGGAAGCACTGGAGTCACG | 4,189,769 – 4,189,750 |  |  |
| 362-kb plasmid | 362-kb-F | CAGTTTGCCATGCCCAGAAC | 328,783 – 328,802 | sppA 2 gene | 106 bp |
|  | 362-kb-R | CTCCGACCCCACAACATCC | 328,888 – 328,870 |  |  |
| 4.8-kb plasmid | 4.8-kb-F | TCAAGCATGGCAGGATCAGG | 4,293 - 4,312 | Kpn23412_5492 | 100 bp |
|  | 4.8-kb-R | ACAGGCCATGCTCCTTCTTG | 4,392 - 4,373 |  |  |
| 3.8-kb plasmid | 3.8-kb-F | TCTCGCATGTGTATCCTTCAGG | 2,823 - 2,844 | Kpn23412_5496 | 103 bp |
|  | 3.8-kb-R | TCGCTGGATGTTAAGGAAGATGG | 2,925 - 2,903 |  |  |

**Supplementary Table S1.** Primers used for qPCR to determine plasmid copy numbers.
